# A Guide to Inverse Kinematic Marker-Guided Rotoscoping Using IK Solvers

**DOI:** 10.1093/iob/obac002

**Published:** 2022-01-27

**Authors:** Ashleigh L A Wiseman, Oliver E Demuth, John R Hutchinson

**Affiliations:** Structure and Motion Laboratory, Department of Comparative Biomedical Sciences, Royal Veterinary College, University of London, London NW1 0TU, UK; McDonald Institute for Archaeological Research, University of Cambridge, Cambridge, CB2 1TN, UK; Structure and Motion Laboratory, Department of Comparative Biomedical Sciences, Royal Veterinary College, University of London, London NW1 0TU, UK; Department of Earth Sciences, University of Cambridge, Cambridge CB2 1TN, UK; Structure and Motion Laboratory, Department of Comparative Biomedical Sciences, Royal Veterinary College, University of London, London NW1 0TU, UK

## Abstract

X-ray Reconstruction of Moving Morphology (XROMM) permits researchers to see beneath the skin, usually to see musculoskeletal movements. These movements can be tracked and later used to provide information regarding the mechanics of movement. Here, we discuss “*IK marker-guided rotoscoping*”—a method that combines inverse kinematic solvers with that of traditional scientific rotoscoping methods to quickly and efficiently overlay 3D bone geometries with the X-ray shadows from XROMM data. We use a case study of three Nile crocodiles’ (*Crocodylus niloticus*) forelimbs and hindlimbs to evaluate this method. Within these limbs, different marker configurations were used: some configurations had six markers, others had five markers, and all forelimb data only had three markers. To evaluate IK marker-guided rotoscoping, we systematically remove markers in the six-marker configuration and then test the magnitudes of deviation in translations and rotations of the rigged setup with fewer markers versus those of the six-marker configuration. We establish that IK marker-guided rotoscoping is a suitable method for “salvaging” data that may have too few markers.

## German

Die Röntgen-Rekonstruktion sich bewegender Morphologie (XROMM) bietet den Forschern eine Möglichkeit, unter die Haut zu blicken, in der Regel, um Bewegungsabläufe des Bewegungsapparates nachzuvollziehen. Diese Bewegungen können erfasst und später verwendet werden, um Informationen über die Funktionsweise der Bewegungen zu erhalten. Hier beschreiben wir das “IK marker-guided Rotoscoping” - eine Methode, die Lösungen aus der inversen Kinematik mit traditionellen wissenschaftlichen Rotoskopiemethoden kombiniert, um 3D-Knochengeometrien schnell und effizient mit Röntgenschatten aus XROMM-Daten zu überlagern. Diese Methode wird anhand einer Fallstudie an den Vorder- und Hintergliedmaßen von drei Nilkrokodilen (*Crocodylus niloticus*) bewertet. Für die einzelnen Gliedmaßen wurden unterschiedliche Markerkonfigurationen verwendet: Einige Konfigurationen der Hintergliedmassen enthielten sechs Marker, andere fünf Marker, sämtliche Vordergliedmaßen enthielten jeweils nur drei Marker. Um das IK marker-guided Rotoscoping zu evaluieren, entfernten wir systematisch Marker in der Sechs-Marker-Konfiguration und verglichen dann die Abweichungen von Translationen und Rotationen der mit weniger Markern ausgestatteten Setups mit denjenigen der Sechs-Marker-Konfiguration. Wir konnten aufzeigen, dass das IK marker-guided Rotoscoping eine geeignete Methode ist, um zusätzliche Daten zu gewinnen, wenn die Anzahl enthaltener Marker anderweitig unzureichend ist.

## Italian

X-ray Reconstruction of Moving Morphology (XROMM) consente a ricercatori di vedere sotto la pelle, solitamente per osservare movimenti muscoloscheletrici. Questi movimenti possono essere tracciati per poi ottenere informazioni sulle meccaniche del movimento. In questo articolo discutiamo ‘IK marker-guided rotoscoping’ - un metodo che combina inverse kinematic solvers con metodi scientifici tradizionali di rotoscoping per sovrapporre modelli geometrici 3D delle ossa in modo rapido ed efficiente utilizzando le ombre a raggi x dai dati XROMM. Per valutare questo metodo usiamo come studio di caso arti anteriori e posteriori di tre coccodrilli del Nilo (*Crocodylus niloticus*). Tra questi arti, sono state usate diverse configurazioni per i marker: alcune configurazioni avevano 6 marker, altre 5 e tutti gli arti anteriori avevano 3 marker. Per valutare ‘IK marker-guided rotoscoping', sistematicamente rimuoviamo marker dalla configurazione che usava 6 marker e poi testiamo la magnitudine della deviazione in traslazione e rotazione tra il modello con meno marker e quello originale da 6. Stabiliamo che ‘IK marker-guided rotoscoping' è un metodo adatto per ‘salvare' dati che possono contenere troppo pochi marker.

(by Vittorio La Barbera)

## Japanese

X**線動態構築** (XROMM) は筋骨格運動など皮下の観察を可能にする.撮影された筋骨格運動は運動機構に関する知見をもたらす.本研究では, “IK (逆運動学) マーカー・ロトスコープ法” - 逆運動学ソルバーと元来の科学的ロトスコープ法の組合せにより, 迅速かつ効果的にD骨ジオメトリーとXROMMから得たX線陰影を重ね合せた.本手法は, ナイルワニ (*Crocodylus niloticus*) の前後肢を用いた事例研究により評価された.異なる前後肢のマーカー構成 (5もしくは6マーカー, 全ての前肢は3マーカー) が用いられた.IKマーカー・ロトスコープ法を評価するため, 6マーカー構成から体系的にマーカーを取除き, 少ないマーカー構成と6マーカー構成で組立モデルの並進・回転方向のずれ度合を比べた.その結果, IKマーカー・ロトスコープ法は, 少ないマーカー構成から得られたデータを“救出”するのに適した手法であることが分かった.

(by Masaya Iijima)

## French

La reconstruction par rayons X de la morphologie en mouvement (XROMM) permet aux chercheurs d'observer les structures internes d'un organisme en mouvement, notamment au niveau musculo-squelettique. Ces mouvements peuvent être suivis à l'aide de marqueurs, renseignant ainsi sur la mécanique des mouvements de l'organisme étudié. Nous présentons ici la méthode  ‘IK marker-guided rotoscoping’ qui combine des solveurs de cinématique inverse (IK) et des méthodes de rotoscopie scientifique pour superposer efficacement et de manière fiable des os en 3D avec les données obtenues par XROMM. Afin d'estimer la précision de cette méthode, nous l'avons appliquée sur un échantillon composé de membres antérieurs et postérieurs de trois crocodiles du Nil (*Crocodylus niloticus*) en utilisant trois configurations différentes : certaines comprenaient six marqueurs, d'autres cinq, tandis que toutes les configurations sur le membre antérieur comprenaient trois marqueurs. Nous avons ensuite retiré des marqueurs dans toutes les configurations comprenant six marqueurs afin de vérifier si cette différence avait un impact sur les mouvements de translations et de rotations obtenus respectivement avec ces deux configurations. Nous démontrons ainsi qu'il est possible d’étudier des données incomplètes, et donc n'offrant que très peu de marqueurs, en utilisant la méthode ‘IK marker-guided rotoscoping’.

(by Romain Pintore)

## Introduction

X-ray Reconstruction of Moving Morphology (XROMM) has permitted biologists and biomechanists to visualize and measure *in vivo* musculoskeletal movement and *ex vivo* capacities of an organism with precision and accuracy (e.g., Brainerd et al. 2010; Gatesy et al. 2010; Nyakatura and Fischer 2010; Stefen et al. 2011; Baier and Gatesy, 2013; Arnold et al. 2014; Kambic et al. 2014; Nyakatura et al. 2014; Camp et al. 2015; Brainerd et al. 2016; Panagiotopoulou et al. 2016; Brocklehurst et al. 2017; Kambic et al. 2017; Fischer et al. 2018; Capano et al. 2019; Bhullar et al. 2019; Nyakatura et al. 2019; van Meer et al. 2019; Manafzadeh 2020; Tsai et al. 2020; Turner et al. 2020; Bishop et al. 2021a; Jones et al. 2021; Manafzadeh and Gatesy 2021; Manafzadeh et al. 2021; Wiseman et al. 2021; Turner and Gatesy 2021; Wheatley et al. 2021; Regnault et al. 2021). XROMM captures movement data using biplanar X-ray videos, which is then integrated with 3D scan data consisting of skeletal elements of the same specimen. The result is a 3D animation of an organism's movement from which biomechanical parameters can be extracted and analyzed (e.g., joint angles; Kambic et al. 2014; Wiseman et al. 2021). XROMM is facilitated by either marker-based (e.g., Brainerd et al. 2010) or markerless (e.g., Gatesy et al. 2010) approaches, in which the marker-based approach requires the researcher to place radiopaque markers (e.g., Menegaz et al. 2015) on anatomical landmarks, which assists in aligning the X-ray captured images with 3D scan data of the same specimen (typically bones). The markers can either be surgically implanted into the organism (e.g., Kambic et al. 2014; Manafzadeh 2020), or be affixed to the skin (e.g., Baier & Gatesy 2013; Hatala et al. 2018; Turner et al. 2020).

A marker-based approach requires the use of dedicated software for (1) correcting image distortion created by fluoroscopic image intensifiers, (2) camera calibration, and (3) marker tracking through space and time (Knorlein et al. 2016). Such software has also been used for the creation of rigid-body elements whereby if three or more markers are present in the same bone (e.g., an animal's thigh/femur), then a rigid body can be created in which the 3D bone element can be animated relative to the rigid body (e.g., Brainerd et al. 2010; Knorlein et al. 2016; Manafzadeh 2020). The freely available, multiple-platform software XMALab, which excels in precision and accuracy (Knorlein et al. 2016), has been developed to process XROMM data, and is recommended as a “best practise” approach by Manafzadeh (2020). XMALab can be combined with other software packages (e.g., DeepLabCut) to facilitate and accelerate the tracking of repeated cyclical movements within limited parameters (Laurence-Chasen et al. 2020). However, few studies have discussed a generalized approach to post-processing of such tracked data (i.e., after data collection and the tracking of markers in XMALab) to standardize the way in which scientists use movement data by combining the XROMM data with 3D bones (i.e., polygonal meshes) of the same specimen. While most studies use Autodesk Maya, San Rafael, CA for post-processing, the manner in which the bones are aligned with the X-ray shadows is not standardized. This is partly because such a range of applications for XROMM data exists that a need for a “one-size-fits-all” approach precludes standardization, but for repeatable cyclical movements, it could be preferable to adopt a standardized approach. While some studies (e.g., Kambic et al. 2014; Bishop et al. 2021b) highlighted the need for standardizing the way in which joint axes are created, there currently are many different ways in which the 3D scanned bones come to align with the X-ray images (see: Table 1). Here we propose a quick and efficient method of the bone alignment procedure that is applicable to XROMM-captured limb-only movement, primarily for scenarios in which the movement data will be used in musculoskeletal modeling whereby each given joint has reduced degrees of freedom (DOFs) (see below). Whilst the inverse kinematic (IK) solver is only tested here on limbs, the method may be applicable with further investigation to other skeletal bodies, such as digits, or other musculoskeletal movements.

**Table 1:** Details of the advantages and disadvantages of scientific rotoscoping, IK solver (markerless and marker-guided), and marker-based XROMM methods. Further details regarding IK set-up are in Supplementary Information 2.

	Advantages	Disadvantages
**Scientific rotoscoping**	✓ Precise anatomical fidelity✓ No loss of degrees of freedoms✓ Markerless or marker-based	× Slow processing time per trial (days)× Hand alignment method; difficult to master and requires greater training× Manual animation
**(Markerless) IK rotoscoping**	✓ No markers necessary✓ Quick animation per trial✓ Allows virtual experiments	× Reduction of anatomical accuracy× Assumptions about joint degrees of freedom× Unnatural joint poses possible× Loss of certain degrees of freedom, dependent upon research question/feasibility× Manual animation
**IK marker-guided rotoscoping**	✓ Quick set-up per focal specimen (30 min)✓ Quick animation per trial (10 to 30 min dependent on additional rotoscoping required due to number of tracked markers; see Methods)✓ Marker-guided alignment; less input training✓ Adaptable to the minimum number of markers required if markers become “lost”✓ Extrapolation of ‘marker-bracketed’ segments✓ Semi- to fully automated (depending on the number of markers)	× Reduction of anatomical accuracy× Loss of certain degrees of freedom, dependent upon research question/feasibility× Marker-only method× Not suitable if number of markers are less than 3 in the whole limb/desired body segment
**Marker-based XROMM**	✓ Precise anatomical fidelity✓ No loss of degrees of freedoms✓ Rigid body transforms can be used to extend the number of usable frames if one marker is lost in a single frame✓ Fully automated	× Rigid bodies can only be processed if all markers are visible in at least one of the X-ray fields of view, thus reducing the number of frames that are trackable

There are two popular methods to align the 3D bones with the X-ray images in a markerless environment. The first is called “scientific rotoscoping” (e.g., Gatesy et al. 2010; Cieri et al. 2020; Turner et al. 2020; Turner and Gatesy 2021), which can use Autoscoper methods (i.e., Miranda et al. 2011; Akhbari et al. 2020), and the second is using an IK solver (e.g., Watt and Watt 1992; Nicolas et al. 2007; Nyakatura et al. 2019; Nyakatura & Demuth, 2019; Zwafing et al. 2021), although the latter typically can be marker-guided. The goal of both scientific rotoscoping and IK solvers is to accurately align the 3D bones with the bone shadows of the X-ray images (Miranda et al. 2011). After this alignment, joint angles can be extracted, which can be used to animate a (musculo)skeletal model of the organism. The model can then be used to estimate biomechanical parameters of the organism, such as joint moments, moment arms, and muscular mechanics (e.g., Nyakatura and Fischer 2010; Bonnan et al. 2016; Orsbon et al. 2018; Bhullar et al. 2019; Olsen et al. 2019; van Meer et al. 2019; Akhbari et al. 2020; Bishop et al. 2021a; Wiseman et al. 2021). If major discrepancies are present between the 3D bones and X-ray images, then any biomechanical outputs are potentially flawed. Slight deviations in resulting joint angles may be of little concern because these become somewhat redundant when rotoscoped data are filtered and smoothed prior to implementation in biomechanical software (e.g., Bishop et al. 2021a; Wiseman et al. 2021), such as OpenSim (Delp et al. 2007).

The method (scientific rotoscoping versus IK solvers) should reflect the research goals. For example, biomechanical studies using musculoskeletal models to investigate parameters such as joint moments, moment arms, and/or muscular mechanics typically only include three DOFs at the most proximal joint, with all distal joints typically composed of a single DOF; just flexion–extension (see: Delp et al. 1990; Seth et al. 2016; Wiseman et al. 2021; Bishop et al. 2021a,  2021b; but also see: Kambic et al. 2014; Wheatley et al. 2021). This is in contrast to other studies investigating range of motion around a given joint whereby all six DOFs are preferential (i.e., Manafzadeh and Gatesy 2021) and are incorporated into scientific rotoscoping approaches, but not in IK solver approaches. Six DOFs in the former musculoskeletal modeling scenario may not be necessary or even feasible for the research question and method at hand. Selection of the rotoscoping method should be complementary to the research goals and tools, and—in scenarios whereby DOFs are planned to be limited in the musculoskeletal model—this should also be incorporated into the rotoscoping setup. This may lead to some loss of anatomical fidelity, which the study should acknowledge as a limitation (i.e., loss of DOFs; see discussion in Kambic et al. 2014). Both methods are described below.

### Scientific Rotoscoping

**Fig. 1:**
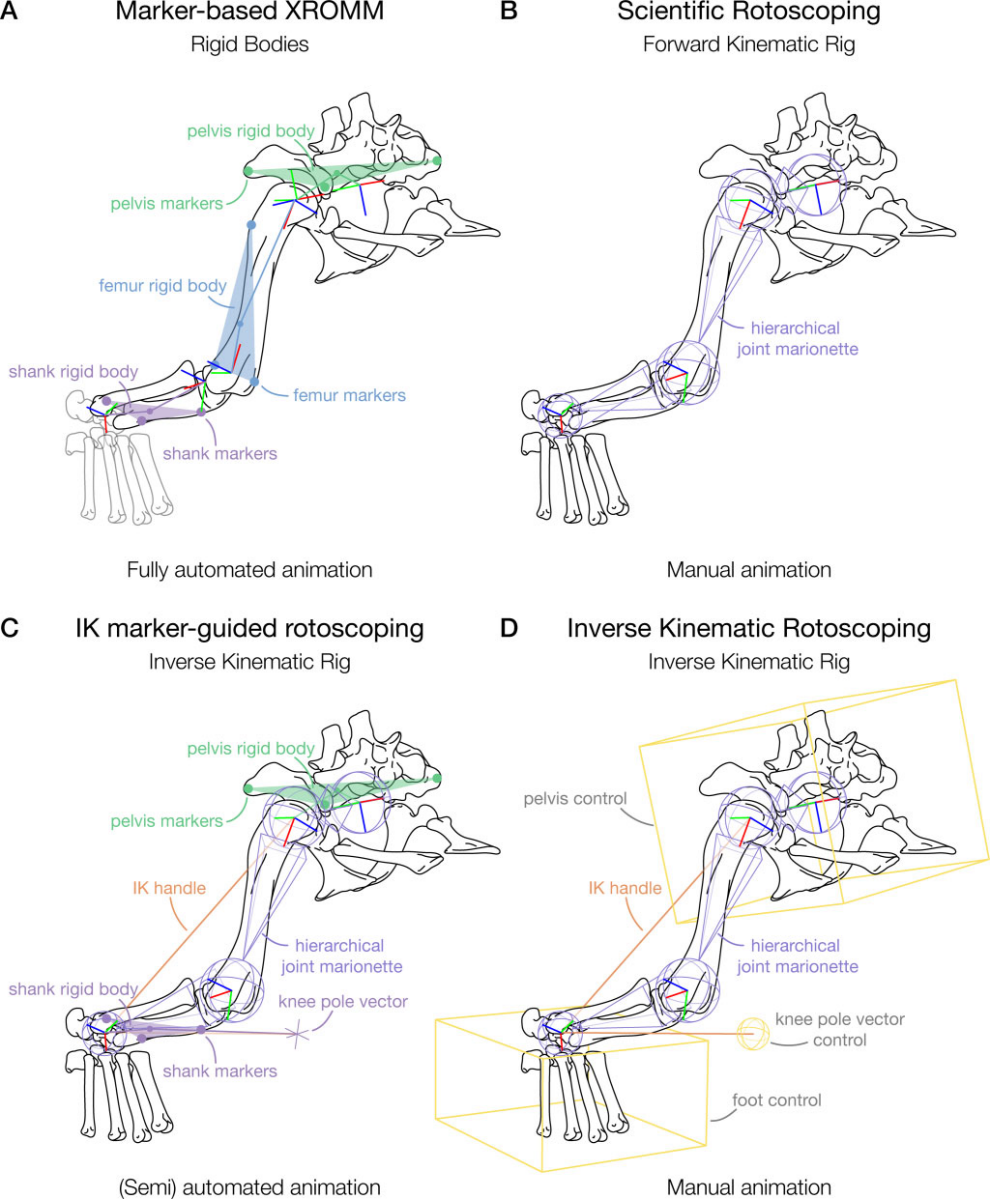
Overview of the different setups to calculate joint motion from kinematic data; using a (right) hindlimb example for a crocodylian in Maya software (Autodesk, San Rafael, CA). (A) Rigid body setup of marker-based XROMM, in which the bones are animated through the markers and joint motion is backcalculated from the relative orientation and position of its JCS and ACS (see Brainerd et al. 2010; Kambic et al. 2014; Knorlein et al. 2016; Manafzadeh 2020). The shank is assumed to be a rigid body here for simplicity; in reality it is not as the tibia/astragalus and fibula rotate relative to one another. (B) Forward kinematic joint marionette setup for scientific rotoscoping. The bones are parented to the respective joint objects (purple), which are manually animated to match the X-ray shadow (see Gatesy et al. 2010; Arnold et al. 2014). (C) Setup for IK marker-guided rotoscoping as proposed herein (see Methods). In sum, it is functionally similar to IK rotoscoping, but instead of manually animating control objects, the IK setup is automatically animated through the markers, only needing manual input if the number of markers is not sufficient for full automation (see below). Note, the femur orientation is automatically calculated through the bracketing segments that are marker-guided using an IK solver. (D) IK setup for markerless IK rotoscoping. The control cubes (yellow) are manually animated to position and orient the pelvis and foot, while the rotations of the limb segment are calculated using an IK handle (orange) with a rotate plane IK solver and a control sphere (yellow) for the knee joint (aim object with pole vector constraint, which can either be manually animated or constrained to the foot control box), as applied by Nyakatura et al. (2019), Nyakatura & Demuth (2019), and Zwafing et al. (2021).

To align the 3D bones with X-ray shadows, it is possible to use scientific rotoscoping, which can have high anatomical fidelity by modeling all six DOFs around a given joint (e.g., Gatesy et al. 2010; Brainerd et al. 2010; see: Table 1). This method uses hierarchical marionettes in which virtual joints are used to articulate the 3D bones into position, followed by hand-aligning each 3D bone to their respective bone shadow on the X-ray images (e.g., Sullivan 2010; Nyakatura and Fischer 2010; Nyakatura et al. 2014; Nyakatura et al. 2019; Fig. 1B). This method uses forward kinematics (FK), in which movement is sequentially chain-ordered, with a root to which all motion is linked, descending from parent to child. For example, in a hindlimb model, the pelvis would be the root/parent and the positions and orientations of the hip, knee, and ankle joints (their respective children) would be dependent on movement further up in the chain. To rotate around the ankle joint, first, the pelvis would need to be correctly positioned and oriented, followed by rotation around the hip joint and then the knee joint. Any changes that occur toward the root require downstream correction. Thus, the process can be slow and time-consuming for an inexperienced user (Gatesy et al. 2010) and require many hours (or even days) of processing to animate just a single XROMM-captured trial. However, as an individual repeatedly rotoscopes trials, accuracy and processing times will improve with practice. Many studies may capture multiple, repeated trials of the same specimen, which could take a long time for an inexperienced user to process. For example, for just one of the crocodiles included in Wiseman et al.’s (2021) study, a total of 42 separate trials were captured via XROMM in which some of the trials were 20 seconds long, captured at 250 frames /s, totaling 5000 frames per trial. The 3D bones must align with each of these frames. Scientific rotoscoping could take days to align the 3D bones with the XROMM data for just a single trial, although movement can be interpolated (cubic–spline interpolation) between key frames to generate smooth motion and approximate kinematic data (Nyakatura et al. 2014) and speed up the process. In projects that collect large amounts of data, it could easily take up to a year for a novice to complete the rotoscoping stage of a research study, although a more trained individual could process such data more efficiently.

### IK solvers

IK solvers (Watt and Watt 1992; Nicolas et al. 2007) present a more intuitive and faster alternative to the manual positioning and aligning of the skeletal elements. With an IK setup, the user can change translations and rotations of the root joint in the hierarchy without it affecting downstream positioning of the distal joint, while also having the facility to move a downstream joint, which automates the positioning of intermediate joints, thus expediting the whole process. For example, a rigged hindlimb would at least have a hip, knee, and ankle joint. If the ankle joint was rotated and translated into position and then the user noticed that the hip was out of position, changes around the hip would not affect the ankle's position because the IK solver automatically extrapolates the new ankle, knee, and hip rotations. This process, therefore, minimizes repetitive manual manipulation of joints, as can be the case for scientific rotoscoping.

The effectiveness of IK methods is evident in their popular usage for character animation in the entertainment industry (e.g., Agarwala et al. 2004) and is even the primary factor facilitating computer-generated imagery (CGI) in films (e.g., Hennessey 2017). Each position and orientation of the bones is automatically calculated based on the relative position and orientation of control points (see Gatesy et al. 2010). These control points can either be manually positioned and animated (Nyakatura et al. 2019; Nyakatura & Demuth 2019; Zwafing et al. 2021; Fig. 1A) or combined with markers to guide their position and orientation (Wiseman et al. 2021; Fig. 1C). If using an IK solver, the time to process the crocodile XROMM data from Wiseman et al.’s (2021) study would be appreciably reduced. One IK rig can be established for each individual specimen in around 30 minutes from which an infinite number of trials can be animated per rigged specimen. The IK rig is guided into position by the tracked markers (see Supplementary Information 2). Afterward, the tracked 3D coordinates of each trial (i.e., 42 trials for one specimen from Wiseman et al. (2021) are used to animate the rig, resulting in a processing time of each trial of between 10 and 30 min in Maya [this time excludes processing in XMALab]). Longer processing times only occur if additional rotoscoping is required because it cannot be guided by the markers (i.e., see Supplementary Information 2). In practise, as many as 15–20 trials can be completed in a single work day if one factors in the time spent saving outputs, and in just 1 week, all trials for a specimen can be post-processed using an IK solver. However, we stress that the estimated time provided here is based on our own data, and that processing time can vary due to a wide variety of factors such as the number of markers present (see methods below), the placement of markers, and X-ray image quality. Nevertheless, we advocate that IK solvers are quicker than FK-based methods.

### Advantages and disadvantages of IK

It is important to stress that both rotoscoping and IK solvers have so-called “trade-offs” when aligning the 3D bones with the tracked markers. Rotoscoping offers better anatomical fidelity, but IK solvers are quicker while maintaining some accurate anatomical representation, and reducing those DOFs that are not pertinent to the research question or feasible under other constraints (Table 1). A researcher must weigh the advantages and disadvantages of both methods with respect to their research question. Markerless IK (Fig. 1A) allows for fast approximation of kinematic data and additional virtual experiments to be conducted (see Nyakatura et al. 2019; Nyakatura and Demuth 2019; Zwafing et al. 2021), and while scientific rotoscoping (Fig. 1B) is bound to the FK framework, it allows capturing precise anatomical fidelity. Marker-based XROMM (Fig. 1D) is dependent on the visibility of the markers for the creation of rigid bodies. If each bone of interest has at least three (fixed) markers (which must be visible in at least one camera at all times), marker-based XROMM allows reconstruction of movement with most accuracy and precision. IK solvers allow extrapolation of the movement when markers are lost or invisible in parts of a trial (Camargo et al. 2020). If a researcher has marker-based data with suboptimal marker placement (i.e., less than three consistent markers per bone of interest) and only requires limited DOFs to be modeled around each given joint, then the speediness of an IK solver, combined with its adaptability, is suitable (see Case study). While the IK solver has only been tested on (proximal) limbs here, the method can be applied to other skeletal bodies (e.g., fingers and toes or multi-segmental rib movement in reptiles, or even extended to spline IKs for vertebral movement).

Both scientific rotoscoping (see Turner et al. 2020; Turner and Gatesy 2021) and the IK solver can be marker-guided, but the IK solver is not reliant upon a specific number of markers to automate the bones into position, permitting this method to be used in a variety of circumstances, albeit with reduced anatomical fidelity (Table 1). While the work of Brainerd et al. (2010) encouraged scientists to place a minimum of three markers per desired body segment in designated positions, this might not always be possible. Nevertheless, there are scenarios in which data collection does not go to plan: inexperienced users may accidentally use fewer markers, or ethical or practical restrictions may impede surgical implantation, alongside veterinary care of the animal. Furthermore, markers that are placed too far from the bone (either intentionally or via surgical mistakes) in surrounding soft tissue may “travel” in the body during the animal's recovery period, and the presence of such “lost” markers may not be discovered until marker tracking begins after data collection. Then, what can a researcher do if they discover that they do not have an adequate number of markers (i.e., three or more per segment) in their dataset to create rigid bodies to assist in 3D bone alignment? If the movement data will subsequently be used in musculoskeletal modeling that will have reduced DOFs as part of the research study design, an IK solver is a suitable solution to track XROMM data because the method is adaptable dependent on the number of markers present within a limb, although a minimum of three markers in the limb are still required (i.e., this could be two markers in the pelvis and one marker in the shank segment; Supplementary Information 2) to allow (partial) automation of the animation process. The method remains untested on other skeletal elements, but could readily be adapted for use in other multi-segmental parts of a body, as long as the underlying assumptions are acknowledged (i.e., reduction of DOFs).

Here, we propose a guide for aligning 3D scanned bones with tracked XROMM data using an IK rig in the software package Maya (Autodesk, San Rafael, CA), similar to markerless XROMM data by Nyakatura et al. (2019) for *Orobates*, and Nyakatura and Demuth (2019) and Zwafing et al. (2021) for caiman (see also: Watt and Watt 1992; Nicolas et al. 2007). However, the rig is guided through markers to improve kinematic data capture and data throughput via (partial) automation. We discuss three different rig creations, each one dependent on the number of surgically implanted markers and the relevant changes to the rig to accommodate “lost” markers for suboptimal and limited marker placement (i.e., fewer than three per segment (see Brainerd et al. 2010)). The temporary loss of additional markers (i.e., if they fall outside of the field of view) can be overcome to extend the duration of captured trials, although this is also possible using rigid bodies if one marker is still present in at least one of the fields of view. In contrast to fully marker-based XROMM, our approach also allows extrapolating the orientation of body segments that lack markers themselves, assuming that marker-guided segments bracket them, for example, the orientation and location of the femur when markers inform the pelvis and shank positions and orientations (see below). The goal of our study is to offer a clear and concise practical guide to determining *in vivo* skeletal animation from XROMM data using IK rigs. Here, our rigs have been created using a case study of the fore- and hindlimbs of three *Crocodylus niloticus* (Nile crocodile) specimens (see Wiseman et al. 2021), but the protocol is applicable to other tetrapods, as evidenced by IK solvers used by other studies (e.g., Nicolas et al. 2007; Nyakatura et al. 2019; Nyakatura and Demuth 2019; Zwafing et al. 2021).

## Case study: Nile crocodile fore- and hindlimbs

### Materials and methods

We created IK rigs for three female, juvenile Nile crocodiles (*Crocodylus niloticus* Laurenti 1768). All experimental protocols were conducted in the Structure and Motion Laboratory of the Royal Veterinary College, via prior approval by the College's Ethics and Welfare Committee (approval number 20160089 N) and under a project license (P0806ABAD) granted by the Home Office (United Kingdom). Details of specimen acquisition, surgical procedures, animal husbandry and welfare, and XROMM experimentation for these same specimens have previously been discussed (Cuff et al. 2019; Wiseman et al. 2021) and are repeated in Supplementary Information 1.

### Marker placement in the Nile crocodile limbs

**Fig. 2:**
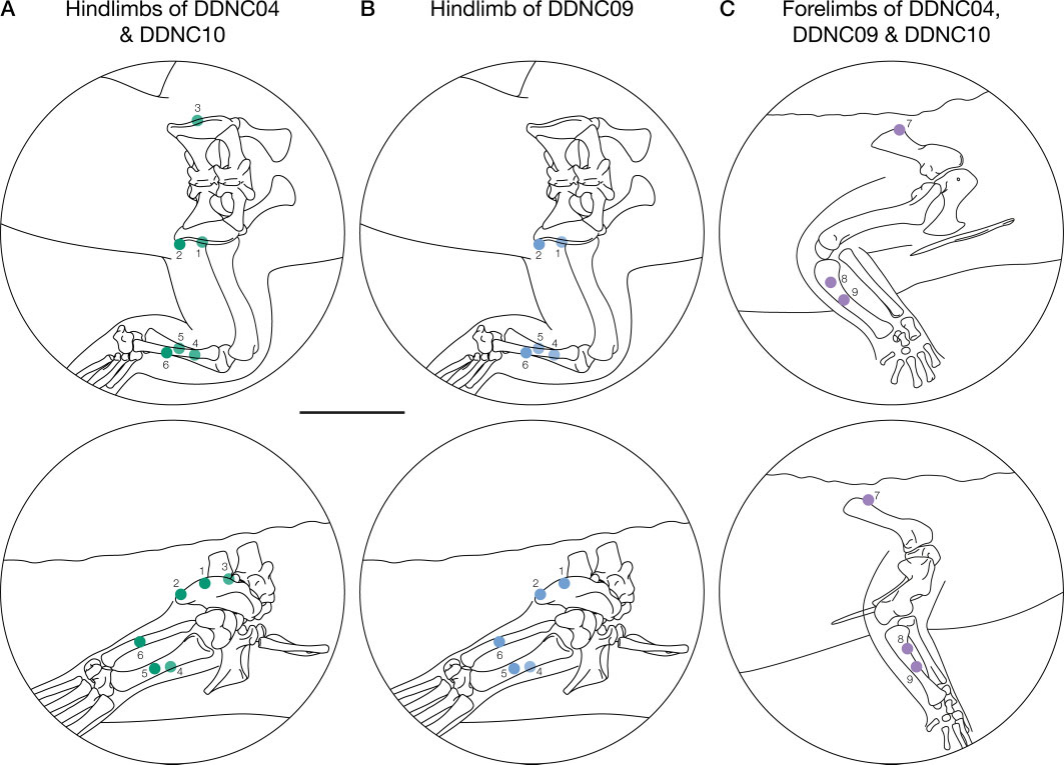
Marker placement in the three Nile crocodile specimens. (A) Six markers in the hindlimb of DDNC04 and DDNC10: three on the pelvis and three on the shank. (B) Five markers on the hindlimb of DDNC09; marker 3 was “lost” in this specimen. (C) Forelimb setup of three crocodiles with three markers; one on the scapula and two on the ulna. All drawings are based on DDNC04 and the bead placement is exemplary for the other specimens. Scale bar = 5 cm.

In sum, three Nile crocodiles (specimen identification codes: DDNC04, DDNC09, and DDNC10) had markers surgically implanted, via eight incisions measuring ∼1 cm, at various points in the pelvis, hindlimb, pectoral girdle, and forelimb. Marker placement was as follows:

- **DDNC04 and DDNC10**: The first and second markers were inserted into the pelvis on the right cranial and caudal parts of the ilium and the third marker was inserted into the left ilium. The fourth and fifth markers were placed on the lateral right tibia at proximal and distal points along the shaft, and the sixth marker was inserted onto the lateral right fibula about midway along with the shaft (Fig. 2A). The seventh marker was inserted into the dorsal side of the right scapula. The eighth and ninth markers were placed into the lateral right ulna at proximal and distal points along the shaft. In this study, the shank and forearm each was modeled as a singular segmental unit (i.e., tibia-fibula and radius-ulna, respectively).
- **DDNC09**: The first and second markers were inserted into the pelvis on the right cranial and caudal parts of the ilium, and the third marker (left ilium) was “lost” inside the body. The fourth and fifth markers were placed on the lateral right tibia at proximal and distal points along the shaft, and the sixth marker was inserted into the lateral right fibula about midway along with the shaft (Fig. 2B). The markers in the forelimb were placed identical to the other two specimens.

The DDNC04 and DDNC10 hindlimbs both had six markers. The DDNC09 hindlimb had five markers. All specimen forelimbs had only three markers (Fig. 2C).

For all specimens, these surgical sites were chosen based on surgeons’ judgements weighing surgical accessibility (based on prior cadaver-based practice) versus potential impact on surgery duration, animal gait and welfare, and feasibility based on staff experience. Each crocodile had a 7-day recovery period prior to the commencement of experiments. No subjects showed evidence of locomotor impairments during experiments.

### XROMM data capture

During experimentation, the specimens were encouraged to move across a walkway with movement captured via biplanar fluoroscopy (Brainerd et al. 2010; Gatesy et al. 2010). Two BV Libra C-arm systems (Koninklijke Philips N.V., Amsterdam, Netherlands) were used, each composed of a BV 300 generator, F017 tube, and BV 300 collimator and intensifier (22.9 cm diameter), with a source-to-image distance of 99.5 cm. Photron FASTCAM Mini WX50 high-speed digital video cameras (Photron, Tokyo, Japan) recorded the trials at 250 frames /s at 2048 × 2048 pixel resolution, with a shutter speed of 1/750 s. Details on trial speeds and types of movement (i.e., a high walk versus a sprawl) are discussed by Wiseman et al. (2021) and are not repeated nor of relevance here.

XROMM data were processed in XMALab (v.1.5.0; Knorlein et al. 2016), in which the markers were tracked through each of the trials (e.g., Brainerd et al. 2010) and filtered using a low-pass Butterworth filter of 10 Hz and then exported as 3D coordinates. Anatomical and joint coordinate systems (ACSs/JCSs) were established using the shape-fitting procedure outlined by Bishop et al. (2021b) and implemented by Wiseman et al. (2021) for these same specimens. JCSs were established for the hindlimb (pelvis, the right hip joint, the right knee joint, the right ankle joint, and the right third metatarsal joint; the digits were modeled as a single unit) and forelimb (pectoral girdle, both shoulder joints, the right elbow joint, the right wrist joint, and the right third metacarpal joint). The Z-axis was flexion/extension, Y was abduction/adduction (ABAD), and X was long-axis rotation (LAR) (Kambic et al. 2014), with a right-handed coordinate system and X–Y–Z rotation order in Maya. Each crocodile model (i.e., one model for each specimen's hind/forelimb) was set up in the “reference pose,” with all joints extended (i.e., the limb was vertically straightened into an unnatural pose) (e.g., Hutchinson et al. 2005; Bishop et al. 2021a,b; Wiseman et al. 2021). Each model (forelimb and hindlimb) comprised 12 DOFs (all DOFs were set to 0 in the reference pose). For example, in the hindlimb, the DOFs were as follows: three at the hip, one DOF each in the knee, ankle, and (third) metatarsophalangeal (MTP) joints; and six DOFs describing the location and orientation of the pelvis in the global coordinate system. These were considered necessary simplifications in light of planned future simulations (which require some minimization of DOFs) and comparisons to data from fossil archosaurs. These assumptions about DOFs will vary based on study design and other constraints. Furthermore, the IK solver approach is only suitable to such models that have these simplifications. More complex study designs that require greater DOFs should use scientific rotoscoping instead.

After (1) tracking marker placement and (2) establishing the ACSs/JCSs in a model, it is possible to use an IK solver for automated bone alignment/rotoscoping. Refer to Supplementary Information 2 for a detailed step-by-step guide on how to create an IK rig, using the crocodile fore- and hindlimbs as an example.

### IK framework

The establishment of an IK solver is dependent on two stages:

IK rig creation: It is first necessary to collect 3D geometries of the bones and surgically implanted markers of the specimen, usually via CT-scanning. The bones and markers are placed into an anatomical position with the establishment of ACS/JCS (e.g., Baier and Gatesy 2013; Kambic et al. 2014), after which an IK solver approach (Watt and Watt 1992; Nicolas et al. 2007; Nyakatura et al. 2019) is used to permit each of the bones to be automatically animated into position (e.g., Nyakatura et al. 2019; Nyakatura and Demuth 2019). To allow the IK solver to calculate the individual joint rotations, the DOFs of the middle joint (knee and elbow for the hindlimb and forelimb respectively) were restricted to only permit rotation around the flexion/extension (F/E) axis (Z-axis in our case) and the direction of the middle joint could be guided by either a *rotation plane* or a *pole vector* to ensure a single viable solution for the IK solver. The restrictions of the DOFs allowed back-calculation of LAR in the hip/shoulder joint as it was equivalent to restricted ABAD in the knee/elbow joint. However, one could argue that these restrictions of the DOF might have minimal impact on accurately representing kinematic data of tetrapods with a mobile zeugopodium, for which the multi-DOF joints could theoretically be decomposed into multiple single DOF joints. Abduction or adduction of the knee or elbow joint may be physically limited through soft-tissues and could be mostly counteracted through respective hip and shoulder LAR. However, there might be longitudinal sliding of the zeugopodial bones relative to each other in the form of a four-bar linkage, for example, wing folding in birds, which results in ABAD at the elbow joint (see Baier & Gatesy 2013; Stowers et al. 2017; also Manafzadeh et al. 2021), which is problematic for DOF decomposition and kinematic data capture. The LAR of the knee and elbow joints mostly correspond to the LAR of the zeugopodia, for example, movement of the tibia and fibula relative to each other while their proximal ends rotate around the respective femoral condyles (Demuth et al. 2020). However, for other taxa with fused or reduced mobility within the zeugopodium, e.g., avian hindlimbs (see Kambic et al. 2014), or when the zeugopodium is modeled as a single segment, the individual DOFs cannot be decomposed and the limitation of knee and elbow ABAD and LAR through an IK solver might impact kinematic data capture. In our case, these were considered necessary simplifications in light of planned future simulations and will vary depending on research questions. Potential solutions to circumvent these restrictions could involve single-chain IK solvers, which, however, might result in other limitations not covered here.

IK rig animation: The IK rig is dependent on marker-guided XROMM data. An IK solver uses the marker positions as they move through time to guide the *IK handle* and *pole vector* for the distal segment (e.g., the shank or forelimb) and the position and orientation of the proximal segment (e.g., pelvic or pectoral girdle), thus ensuring the articulation of the 3D bones, resulting in each of the limb segments (e.g., in the hindlimb which would be the pelvis, thigh, and shank segments) being locked in an anatomical position throughout the motion, by virtue of simplifying assumptions about joint mobility (e.g., number and types of permissible joint movements). Considering a theoretical situation in which only the femur had markers, FK animations with scientific rotoscoping would be suitable because other body segments (such as the shank) could not be rotoscoped into position using IK marker-guided rotoscoping.

**Fig. 3:**
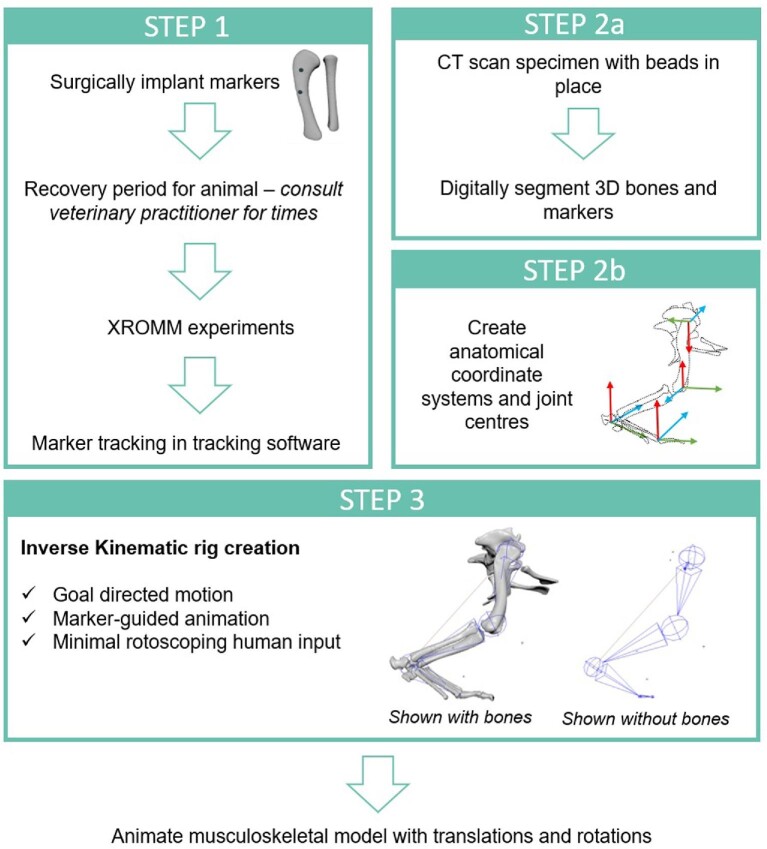
An overflow diagram outlining the three different steps which are vital prior to animating a musculoskeletal model, featuring a Nile crocodile right hindlimb in lateral views. Step one involves preparing the animal for surgery (tibia and fibula bones shown; two markers in the tibia), surgical procedures, and experimentation, including processing experimental data. Step two involves generating 3D scanned data, processing the scans, and creating the anatomical and joint coordinate systems necessary for the IK rig creation, which is step three. Once the IK rig is established and animated, the extracted translations and rotations can be used to animate a musculoskeletal model.

Step-by-step details on how to build the rig and implementation are in Supplementary Information 2. An overview of this process is in Fig. 3. The DDNC04 and DDNC10 rigged hindlimbs were created with six markers in total. The DDNC09 rigged hindlimb had five markers. All rigged forelimbs each only had three markers. Additional rotation was required in the DDNC09 hindlimb to align the pelvis and to accommodate the missing pelvis marker, and additional rotation was required in all forelimbs (Supplementary Information 2; see IK rig evaluation). Additionally, while all rigs successfully tracked the upper segments of each limb (e.g., the pelvis, thigh and shank), manual scientific rotoscoping was required of the more distal segments (e.g., the pes and digits). However, this is a limitation of our own setup in which no markers were placed in the distal segments (e.g., Turner and Gatesy 2021). Ideally, studies should place markers in all segments of interest. However, if—as in our case—this is not feasible, then it is possible to rely upon the orientation and positioning of the more proximal segments to rotoscope the distal segments into position. As the pes and digit segments were FK elements placed hierarchically underneath the IK elements (thigh and shank; see Supplementary Information), they could be manually rotoscoped into position until alignment (i.e., rotate around the ankle joint so that the pes bones align with the shadows). We adopted a simplified approach and only permitted Z-axis rotation (and have acknowledged such limitations previously; Wiseman et al. 2021), but other studies may wish to explore the inclusion of other DOFs.

All rigs were successfully animated using the tracked XROMM markers in which the following joints (right side only) were animated (parent > child hierarchy listed):

Pelvis centre (translation and rotation; six DOFs) > the hip (rotation only) > the knee > the ankle > the MTP joint.

Pectoral girdle centre (translation and rotation; six DOFs) > the shoulder (rotation only) > the elbow > the wrist > the metacarpophalangeal joint.

Rig evaluation is discussed below.

### “Salvaging” missing data using “IK marker-guided rotoscoping”

In the following scenario, six markers have been placed in a limb in consultation with the surgeons, experiments have then concluded, and a researcher begins to track the XROMM data. At this stage, the researcher notices that one marker has become “lost” in the body, resulting in just five visible markers in all trials. How can the orientation of a segment be “salvaged” when one or more markers are missing? With the implementation of an IK solver, the data can still be marker-guided into a relative position and quick processing times are still a possibility. This is feasible if the distances between the joint centers (e.g., the distance between the hip and the knee joint center; the functional length of the femur) are assumed to be constant. This assumption allows an IK setup (with a reduced number of markers) to directly back-calculate the rotation around the axis between the two remaining markers of the first segment if the second segment has all three markers in place. In this circumstance, each joint center in the limb would be used as theoretical markers to estimate the position of the missing markers. Therefore, it is theoretically possible to use an IK setup with just five markers to fully automatically capture the kinematics of the hip and knee joints. While in reality joint spacing, and thus the distances between each respective joint center, may dynamically change (Manafzadeh and Gatesy 2021), these discrepancies might be assumed to be negligible for the purposes of the study. However, if such translations (i.e., sliding) in some joints are present (e.g., the distance between the hip and knee joint does not remain constant throughout the trial due to substantial sliding), then it may be necessary to approximate the joint position of the segment with a missing marker through rotoscoping along the axis between the remaining markers. If further markers become lost, or are even prohibited from implantation upon surgical recommendation, it is still possible to salvage the data to be used with an IK solver. Researchers would need to use additional controls to estimate the position of joint centers and “manually” match 3D bones to the X-ray shadows and approximate their positions and orientations. IK marker-guided rotoscoping has the capability of not only relatively aligning the bones the markers are placed upon, but also easily aligning segments bracketed by them. For full anatomical fidelity, additional manual adjustment is required.

To evaluate the reliability of IK solvers animated with fewer markers, we systematically removed markers of the DDNC04 hindlimb (total markers n = 6) and adjusted the joint marionettes for several cases with different numbers of missing markers, each following the step-by-step guide in Supplementary Information 2. We stress that we only systematically removed markers solely to compare IK solver setups and that we do not compare our data to a scientifically rotoscoped setup because of the differing research goals (e.g., biomechanical simulation versus kinematic animation). Here, we sought to determine how well the data can suit usage in musculoskeletal models that are modeled with limited DOFs (e.g., Bishop et al. 2021b), thereby requiring the rigged setup to have reduced DOFs to replicate the biomechanical modeling software setup. Scientific rotoscoping is more complex and beyond the requirements of such reduced models, and would require further post-processing prior to implementation in biomechanical software.

The following evaluation rigs were created:

1. Evaluation Rig 1, which had five markers, following the marker configuration in the DDNC09 hindlimb (see Fig. 2).
2. Evaluation Rig 2, which had four markers. This rig did not have a comparative configuration (i.e., like Evaluation Rigs 1 and 3) based on our own data, however, we evaluated this scenario to test the effects of four markers on the rig setup and subsequent results, and to describe and illustrate the setup for such cases.
3. Evaluation Rig 3, which had three markers, following the marker configuration of all forelimbs (see Fig. 2).

**Fig. 4:**
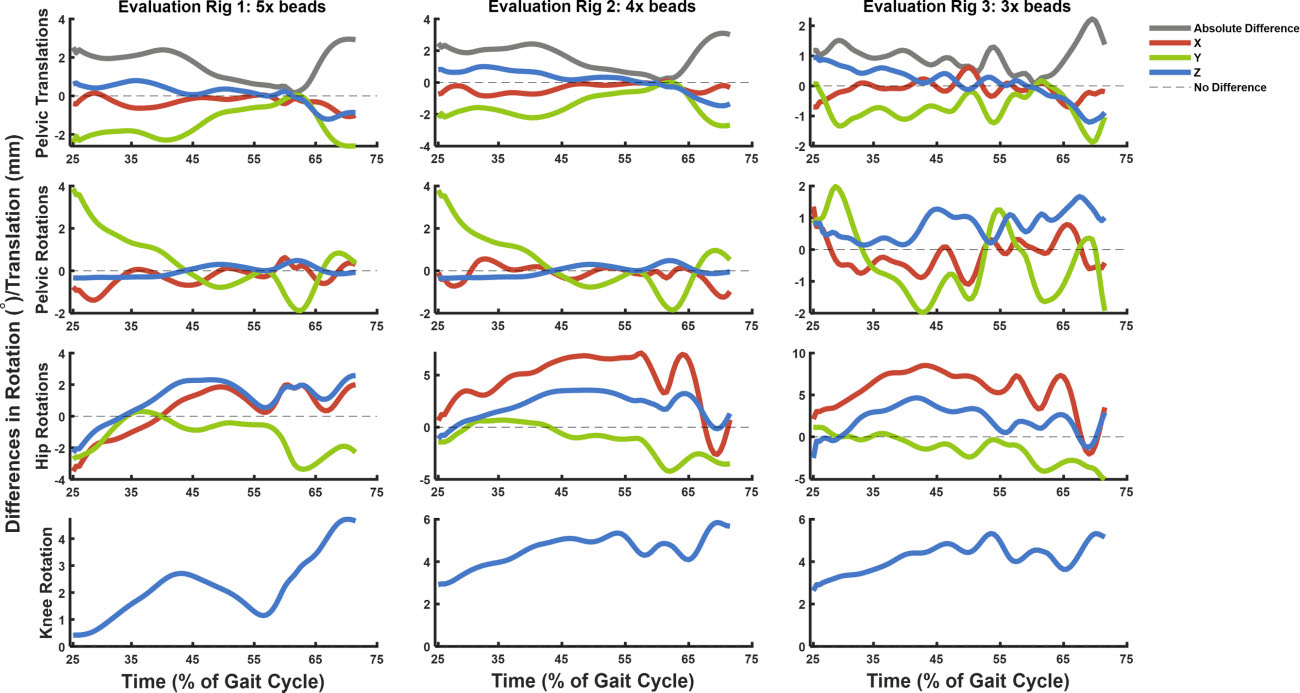
Graphical representation of rig evaluation in which the differences between the original rig and the evaluation rigs are plotted per frame/time step for our sample kinematic dataset. Only Z-axis rotation (flexion/extension) is reported for the knee because we restricted the knee to one DOF. See Table 2 for quantitative comparison and Supplementary Video S1 for visualization of rig deviation.

Evaluation Rig 1 had five markers in which the “lost” marker was the left pelvic marker, identical to the marker configuration in the DDNC09 hindlimb. The two remaining markers in the pelvis were unfortunately placed into the flesh and not directly attached to the bone. Because of this placement away from the bone, the markers moved slightly in relation to the pelvis during limb motion, thus resulting in imprecision and slight deviation of the pelvic position and rotation in comparison with the six-marker configuration, which had one marker embedded directly in the ilium (see Fig. 4). The subsequent removal of additional markers exaggerated this issue and had a “domino-style” effect downstream the joint marionette, with increasing deviations in joint angles in more distally located joints.

We animated one walking trial (n = 93 frames, over a single partial step) using each of these three evaluation rigs and evaluated their individual world space rotations and translations (i.e., in the global reference frame) for the pelvis, and their object space rotations (i.e., local reference frame in relation to their parent body) for the hip and the knee from each rig against the initial captured trial with six markers present. The deviations from the initial joint angles (and positions for the pelvis only) were used to test the accuracy of each rig (each of which had a different number of markers guiding the animation) in relatively tracking the X-ray shadows. The discrepancy in each of the different setups is reported in Fig. 4, Table 2, and visualized in Supplementary Video S1.

**Table 2:** Median differences of the evaluation setups in comparison with the six-marker kinematic data tracking. Translational offsets have been normalized by femur length (58 mm).

	five markers	four markers	three markers
normalized absolute pelvis t offset (%)	3.360 ± 1.409	3.262 ± 1.450	1.695 ± 0.793
normalized pelvis tX (%)	−0.447 ± 0.502	−0.528 ± 0.476	−0.162 ± 0.484
normalized pelvis tY (%)	−3.136 ± 1.369	−2.817 ± 1.324	−1.372 ±0.803
normalized pelvis tZ (%)	0.402 ± 0.957	0.500 ± 1.190	0.291 ± 0.910
pelvis rX (°)	−0.209 ± 0.555	−0.069 ± 0.389	−0.240 ± 0.488
pelvis rY (°)	0.182 ±1.302	0.105 ± 1.274	−0.671 ± 1.079
pelvis rZ (°)	−0.031 ± 0.251	−0.035 ± 0.253	0.826 ± 0.417
hip rX (°)	0.703 ±1.436	5.141 ± 2.519	6.147 ± 2.502
hip rY (°)	−0.799 ± 1.073	−0.980 ± 1.482	−0.999 ± 1.546
hip rZ (°)	1.441 ± 1.201	2.412 ± 1.275	2.314 ± 1.620
knee rZ (°)	2.139 ± 1.149	4.709 ± 0.723	4.378 ± 0.655

Abbreviations: t = translation, r = rotation, X = X-Axis, Y = Y-axis, Z = Z-axis.

The spatial deviations of the pelvis in the evaluation rigs appeared to be relatively minor (see Table 2 and Supplementary Video S1), more so if we consider that the translational offset represented less than 3.5% of femur length (femur length = 58 mm). We are therefore confident that a five-marker setup (as in DDNC09) is suitable to reliably capture the kinematic data for our purposes. Additionally, the marker setups for other potential scenarios also performed well, with only the LAR of the hip joint of the three- and four-marker setups exceeding a 5-degree deviation. Deviations could have been minimized through the placement of the markers directly in bone rather than soft-tissue (e.g., Brainerd et al. 2010). Importantly, the reduction of markers does not impede the tracking of the “middle” segment, discussed below.

### Guided interpolation of the middle segment

The IK setup has a great advantage over the animation of each bone as an individual rigid body with its own world space transformation matrix: It is possible to interpolate the 3D orientation of a bone between two tracked segments even though there are no markers on the middle segment itself. For example, markers placed in the pelvic girdle and in the shank establish the position and orientation of the hip and knee joint and thus allow interpolation of the position and orientation of the femur under the assumption that abduction/adduction of the knee is absent. It is, therefore, not necessary to place markers in the thigh segment as the position and orientation can be interpolated through the bracketing body segments and thus approximated. This applies to both the hind- and forelimbs, in which the middle segment (stylopodium) is, respectively, bracketed by the pelvis and shank or the pectoral girdle and forearm. Thus, six instead of optimally nine markers (i.e., see Manafzadeh 2020 which placed markers on cadavers, not live animals; although see Tsai et al. 2020) were surgically implanted into the hindlimbs of each crocodile, but the implementation of an IK rig permitted us to extract the necessary joint rotations of the hindlimb for our research question regarding a reduced DOF model, through constraining of knee joint rotation axes.

For the forelimb, the implementation of the IK rig permitted us (through minimal rotoscoping) to extract the positions (i.e., translations and rotations) of the pectoral girdle moving through space and the corresponding joint angles of the shoulder, elbow, wrist, and metacarpophalangeal joints-data that can later be used to animate a (musculo)skeletal model to estimate biomechanical parameters of movement. Therefore, it was possible to use IK marker-guided rotoscoping to quickly align the forelimb bones relatively with the X-ray shadows, highlighting the potential of this method.

The evaluation of our marker setups in which we systematically removed markers to ascertain if rotational outputs were consistent established that joint angles remained only slightly affected. The largest discrepancies were measured in hip LAR with a maximum deviation from the reference dataset of 8.5° in the three-marker setup. This deviation is greater than what we would expect to see as standard “noise” error, and thus LAR cannot be modeled with accurate anatomical fidelity in the three-marker setup. Future studies should consider the needs of their respective study if they plan to use a 3-marker setup due to obvious limitations in precise outputs. Nevertheless, we found that fewer markers placed in the limb of an animal still produced kinematic data sufficiently congruent with our reference dataset (see Supplementary Video S1), although the usage of any of such marker setups in future studies should be carefully considered with respect to some loss of anatomical fidelity.

IK solvers are created with reduced DOFs, reflective of their intended subsequent use in musculoskeletal modeling software, for example. The produced data were in line with musculoskeletal modeling, which also tends to have reduced DOFs (e.g., because a full six DOFs could not be made dynamically consistent without modeling all ligaments around all joints to constrain translations; and/or because of limitations on computational power and processing time). We would recommend this method for use in limb-models in which the researcher does not intend to model all six DOFs for each joint. The 3-marker setup is only recommended for use in scenarios where few DOFs are modeled around each joint and/or data must be “salvaged” from poor data collection.

## Concluding remarks

Here, we have presented a new method for animating XROMM data called *IK marker-guided rotoscoping*. This method uses a combination of IK solvers with scientific rotoscoping to quickly and accurately align 3D bone geometries with the shadows of the X-ray images. IK marker-guided rotoscoping is adaptable and easy to implement. While some anatomical fidelity is lost due to a reduction in DOFs, implementation of IK marker-guided rotoscoping offers many advantages, such as improving the post-processing time that a researcher will spend on tracking/animating XROMM data. While we advocate that “more markers are better” (i.e., in an ideal scenario, one would place at least three markers per limb segment), this method also has the great advantage of speedily salvaging missing marker data and should be implemented only in scenarios whereby the end-result is a model with ≤3 DOFs per joint. If a researcher aims to investigate six DOFs around a given joint, they should implement scientific rotoscoping because IK solvers cannot simultaneously capture all six DOFs.

## Supplementary Material

obac002_Supplemental_Files
