# Supplementary material for: A Guide to Inverse Kinematic Marker-Guided Rotoscoping Using IK Solvers
Source: Integr Org Biol. 2022 Jan 27;4(1):obac002. doi: 10.1093/iob/obac002 (PMC8896983; doi:10.1093/iob/obac002)
Supplement: obac002_Supplemental_Files [file obac002_supplemental_files.zip › WisemanEtAl_2022_IKRig_SuppInfo1.docx]

**A guide to inverse kinematic marker-guided rotoscoping using IK solvers**

A.L.A. Wiseman^1*^, O.E. Demuth^1,2*^, and J.R. Hutchinson^1^.

^1^Structure and Motion Laboratory, Comparative Biomedical Sciences, Royal Veterinary College, Hatfield, United Kingdom.

^2^Department of Earth Sciences, University of Cambridge, Cambridge, United Kingdom

*These authors contributed equally to the manuscript.

Email: [alw96@cam.ac.uk](mailto:alw96@cam.ac.uk)

**Supplementary Information 1:**

**Specimen acquisition, surgical procedures and XROMM experimentation**

The following text describes specimen acquisition, surgical procedures and XROMM experimentation for the same specimens used here, which has previously been published (see: Cuff et al. 2019; Wiseman et al. 2021).

*Study animals*

Nile crocodiles were donated from La Ferme Aux Crocodiles (Pierrelatte, France). The crocodiles were housed in the Biological Services Unit, Structure and Motion Laboratory at the Royal Veterinary College, UK, maintained with basking (UV A+B heat lamps), seclusion and swimming habitats as well as enrichment. Temperatures were kept at ~27 °C daytime/~19 °C night-time (12-hour cycle) and with ~70% humidity. In total, there were 10 crocodiles in the enclosures (although only three underwent XROMM experimentation discussed here) and they were kept in groups of 1–5 in walled pens and were fed twice weekly on small vertebrates weighing <10% of their body weight. They were transported to/from the experiments in custom-built crates which were connected directly with the experimental runway, thus minimising human handling.

*Surgical and Experimental Procedures*

Anaesthesia protocol followed Monticelli et al. (2019) as employed by Cuff et al. (2019) for the same specimens used here. We refer to Cuff et al. (2019) for details on the surgical procedure.

**Reference list for Supplementary Information 1.**

Monticelli, P., Ronaldson, H.L., Hutchinson, J.R., Cuff, A.R., d’Ovidio, D. & Adami, C. (2019) Medetomidine-ketamine-sevoflurane anaesthesia in juvenile Nile crocodiles (*Crocodylus niloticus*) undergoing experimental surgery. Veterinary Anaesthesia and Analgesia, 46, 84–89.
