# Supplementary material for: A Guide to Inverse Kinematic Marker-Guided Rotoscoping Using IK Solvers
Source: Integr Org Biol. 2022 Jan 27;4(1):obac002. doi: 10.1093/iob/obac002 (PMC8896983; doi:10.1093/iob/obac002)
Supplement: obac002_Supplemental_Files [file obac002_supplemental_files.zip › WisemanEtAl_2022_IKRig_SuppInfo2.docx]

**A guide to inverse kinematic marker-guided rotoscoping using IK solvers**

A.L.A. Wiseman^1*^, O.E. Demuth^1,2*^, and J.R. Hutchinson^1^.

^1^Structure and Motion Laboratory, Comparative Biomedical Sciences, Royal Veterinary College, Hatfield, United Kingdom.

^2^Department of Earth Sciences, University of Cambridge, Cambridge, United Kingdom

*These authors contributed equally to the manuscript.

Email: [alw96@cam.ac.uk](mailto:alw96@cam.ac.uk)

**Supplementary Information 2:**

**The step-by-step guide of creating an inverse kinematic rig**

An overview of the inverse kinematic (IK) solver process can be found in Figures 1 and 3. A rigged model was created using an IK solver approach (Watt and Watt 1992; Nicolas et al. 2007; Arnold et al. 2014) to permit each of the bones to be automatically rotoscoped into position (e.g., Nyakatura et al. 2019; Nyakatura and Demuth 2019). This approach used the marker positions as they moved through time to guide the articulation of the 3D bones, resulting in the pelvis, thigh and shank segments being locked in anatomical position throughout the motion, by virtue of simplifying assumptions about joint mobility (e.g., number and types of permissible joint movements, i.e. degrees of freedom). This process was repeated for the forelimb. Below we discuss how to create an inverse kinematic (IK) rig in Maya.

**IK rig creation**

Below we provide detailed instructions for the IK rig setups in Autodesk Maya 2019 (Autodesk, San Rafael, CA). The section is sub-divided into four parts with a decreasing number of tracked markers: Six marker setup (DDNC04 and DDNC10 hindlimb), five marker setup (DDNC09 hindlimb and evaluation 1), four marker setup (evaluation 2) and three marker setup (all forelimbs and evaluation 3). The most complex setup with only three tracked beads is illustrated in Supplementary Figure S2 as arranged and annotated in the *Node Editor* and *Outliner*. We additionally provide a Maya 2019 (.ma) file containing the setups of each of the IK rigs described below, which may be used as a template for future study setups. We advise the reader to explore the *Node Editor* of each setup to gain a better understanding of the setup’s constraints, relationships and implementation. In the Maya file, the implanted beads are keyed for several frames based on experimental data from Wiseman et al. (2021) to illustrate how the bead position influences the joint movement. Simultaneous positions for all beads are available only between frames 2328-2420. For all other frames, one or multiple beads were not within the capture area of the biplanar X-ray and thus were not tracked. The beads are keyed in the reference position (straightened limb) at frame 0 and we encourage the reader to navigate to frame 0 to explore the non-tracked IK rig setup.

**Six marker IK rig setups (DDNC04 and DDNC10 hindlimbs):**

- *Maya joint marionette creation:* After the ACSs and JCSs have been established (see: Kambic et al. 2014; Bishop et al. 2021b; Wiseman et al. 2021), a hierarchical joint ‘marionette’ (forward kinematic rig) was created (Gatesy et al. 2010; Arnold et al. 2014) based on the location and direction of the ACSs. The hierarchical order of the forward kinematic (FK) rig was as follows: Pelvis > Hip > Knee > Ankle > MTP > Toe_Tip. The limb was straightened out into the reference pose with a fully extended limb pointing ventrally (Hutchinson et al. 2005), for which the joint Orient of the hierarchical joints was set to (0/0/0) > (90/-90/0) > (180/0/0) > (0/0/0) > (180/0/0) > (0/0/0) and all joint rotations to 0 (see: IK rig set up section below for further details). The *joint Orient* settings enabled the desired joint behaviour and joint rotations to follow the convention of a right-hand rule for counter-clockwise positive rotation (e.g. Sullivan 2007; Kambic et al. 2014).
- To establish the correct relative spatial position of the implanted markers used for XROMM tracking to the Maya joints in the reference pose, the markers were temporarily hierarchically parented underneath the bones from the CT scan dataset before rig creation, thus ensuring that the relative position of the markers to the bones (and joint centres) remained unchanged during rig manipulation. The markers were unparented after the limb was straightened into the reference pose. The global position of each marker in this specific position in the world coordinate system thus represented the fully straightened limb in the reference pose. To save the position of the markers and the reference pose for the inverse kinematic (IK) rig (see below) their position was keyed at frame 0.
- *IK rig set up:* To transform the FK rig into an IK rig, an *IK Handle* with a Rotate-Plane Solver was created from the hip joint to the ankle joint. To help the *IK Handle* to recognize which way the knee flexes, the joint Orient was changed to 0.02⁰ (the lowest possible value recognisable by the software during rig creation; the value is negligible) for the Z-axis in the knee joint, which has no effect on the results but is a technical necessity in the set-up. Otherwise, the IK handle was unable to determine the direction of movement, which is a limitation of the IK solver.
- Three ‘locators’, i.e. helper objects that represent 3D coordinates in space, were created to translate the movements of the markers into skeletal movement of the crocodiles. The first locator, named Pelvis_Orient_LOC, was used to drive the position and orientation of the pelvis joint. This locator was point constrained to all three pelvis markers (right anterior pelvis, right posterior pelvis and left pelvis) with a weighting of 0.3333 for each marker, thus ensuring that the Pelvis_Orient_LOC was always positioned in the virtual centroid of the triangle between all three pelvis markers. It was then aimed at the right anterior pelvis marker with an *aim vector* of (1/0/0) and an *up vector* of (0/1/0) with the right posterior pelvis marker as *World Up Object* for the *Object Up World Up Type*. This ensured that the orientation of the Pelvis_Orient_LOC was always in relation to a virtual plane through the three pelvis markers; i.e., this defines a rigid body object as triangle, with its corners defined by the position of each marker
- The same procedure was used to create the Shank_Marker_Centroid_LOC. It was point constrained to the tibia markers (proximal tibia and distal tibia) with a weighting of 0.5 each. It was then aimed at the distal tibia marker with an *aim vector* of (1/0/0) and an *up vector* of (0/1/0) with the fibula marker as *World Up Object* for the *Object Up World Up Type*. Thus the body segment representing the shank was constrained in location solely by the tibia, and constrained in orientation only by the fibula marker position. This prevents a situation in which the position of the body segment is influenced by movement of the fibula relative to the tibia.
- A third locator, named Knee_AIM_LOC, was created to drive the rotation of the IK Handle to bring the knee joint in line with the shank markers. First, it was brought into the exact position of the Shank_Marker_Centroid_Loc and then moved cranially by an arbitrary amount (2cm in our case). It was then moved laterally to bring it in line with the XY plane of the knee ACS to counteract the mediolateral offset of the Shank_Marker_Centroid_LOC. This locator was then hierarchically parented underneath the Shank_Marker_Centroid_LOC. This ensured that the position and orientation of the Knee_AIM_LOC was driven by the shank markers and that it was always positioned on a plane characterised by the hip, knee and ankle joint, meaning that knee flexion angles did not influence the relative position of the Knee_AIM_LOC. The previously created *IK Handle* was then constrained to the Shank_Centroid_LOC using a *parent constraint* and to the Knee_AIM_LOC using a *pole vector constraint.*
- No markers were placed in the foot, as detailed pes kinematics or biomechanics were not a focus of our research (e.g., Cuff et al. 2019; Wiseman et al. 2021), so it was only necessary and feasible for this study to rotoscope the metatarsus and digits into anatomical position by changing the angle of the ankle and metatarsophalangeal (MTP) joints whereby only flexion/extension (Z-axis rotation) of these distal joints was permitted. The ankle and MTP joints were manually rotated (i.e., rotoscoped) into position (Gatesy et al. 2010). Because the digits were modelled as one unit, the bony shadows for the digits were not a perfect match, but for the aims of our study, this was not of concern. Future studies that aim to determine pes/digit kinematics (i.e., Turner et al. 2020; Turner and Gatesy 2021) should place markers in the pes segments to incorporate said segments into the IK rig for automated articulation.

This IK setup (Supplementary Figure S1A) enabled us to translate the marker translations into kinematic movement of the crocodile hindlimb matching up with the X-ray shadows of the XROMM data (Supplementary Figure S3). The position and orientation of the pelvis is driven by the pelvis markers, while the orientation and position of the crus is controlled by the shank markers. The orientation of the femur is driven by the relative position and orientation of the crus to the pelvis. The desired LAR of the femur is guaranteed, as the Z-axis of the distal femur ACS is forced to be perpendicular to the shank long-axis by the rig setup.


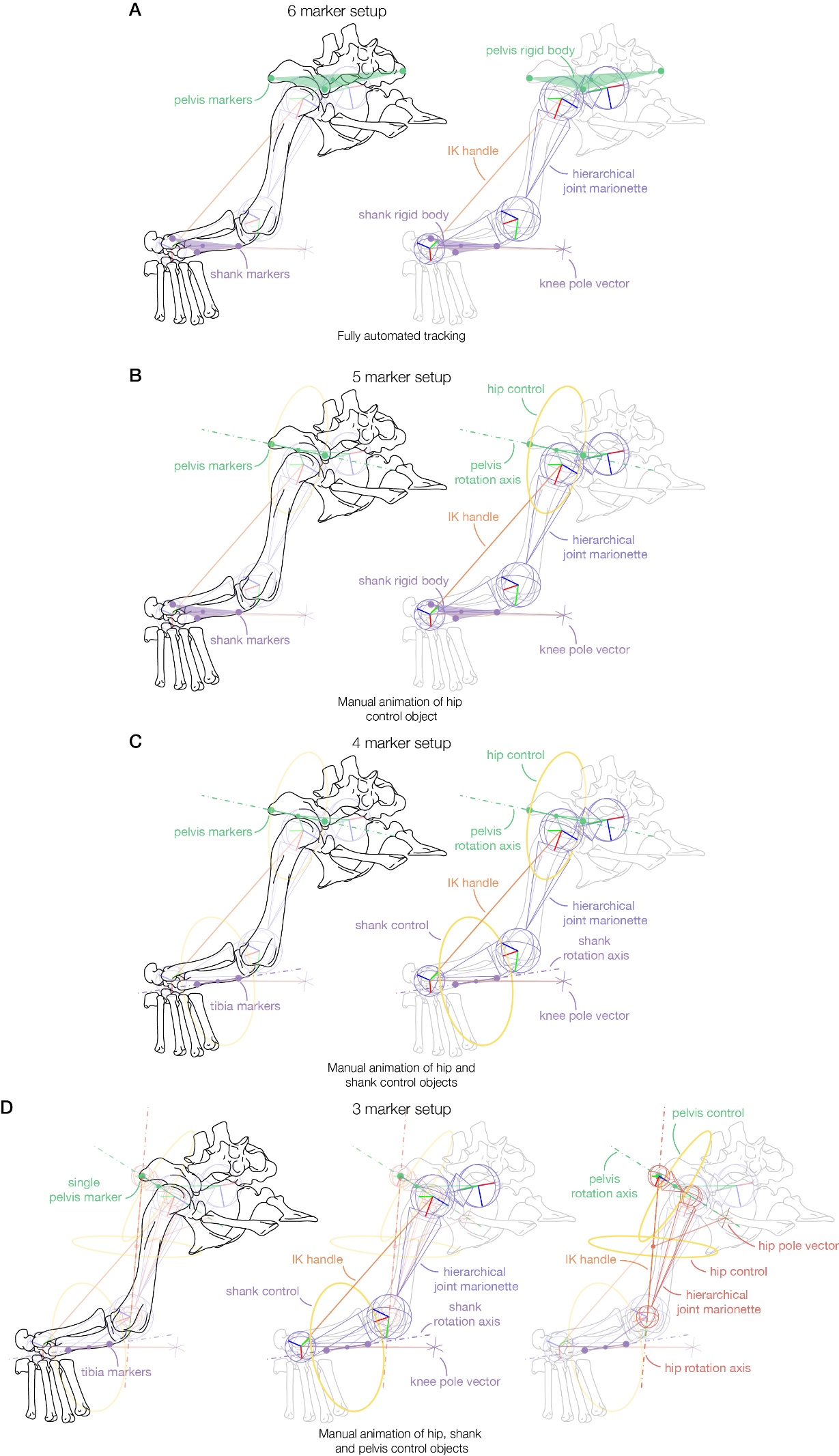


**Supplementary Figure S1.** Schematic illustration of the different IK setups presented in this study. **(A)** Six marker setup as for the hindlimbs of DDNC04 and DDNC10. All motion of the hindlimb is automatically tracked through the marker movement. **(B)** Five marker setup as for the hindlimb of DDNC09 and the evaluation rig 1. The hip control rotates the pelvis and position of the pelvis and acetabulum joint (purple spheres) around the axis between the two green hip markers (marker one and two; green axis), the knee joint automatically follows the movement and aligns the femur with the acetabulum. **(C)** Four marker setup as in the evaluation rig 2. The hip controller acts as in (B), the shank controller rotates the shank, knee and ankle joints, and the knee pole vector locator around the axis between the two tibia markers (marker four and five; purple axis). **(D)** Three marker setup as in the hindlimb evaluation rig 3 and the forelimb setups of all specimens (not illustrated). The shank controller acts identically to (C), but there are two additional controllers and an additional IK rig (salmon coloured joint marionette). The hip controller rotates the acetabulum and hip pole vector around the axis from the knee joint to the single pelvis marker (salmon coloured axis) and thus determines the position of the acetabulum in 3D space. The pelvis controller rotates the pelvis and pelvis joint around the axis from the hip joint to the single pelvis marker (green axis) and thus determines the position and orientation of the pelvis joint. The individual joint rotations are then back-calculated through the IK solver based on the positions and orientations of the pelvis and ankle joints.

**Five marker IK rig setup (DDNC09 hindlimb and Evaluation Rig 1):**

- *Maya joint marionette creation*: It followed the same procedure as DDNC04 and DDNC10 hindlimb setup.
- *IK rig setup:* In the DDNC09 hindlimb, one marker in the pelvis was, unfortunately, ‘lost’ and, as such, only five markers were present. For the Evaluation Rig 2 one pelvic marker was also removed to simulate a trial with a single ‘lost’ marker, both their setup was identical. As in the 6-marker IK setup, the locator for the shank was constrained by its three markers. As for the hip only two markers were present, the locator was point-constrained to the remaining two markers with a weighting of 0.5 each, thus ensuring that it was positioned between both markers. To define the rotation axes the locator needed to be aim-constrained, however, as only two markers were present a world-up object was not available for the pelvis. Thus the pelvis locator was aim-constrained at one of the pelvis markers using one of the shank markers as a substitute world-up object. This defined a plane between the two pelvic markers and one of the shank markers, therefore ensuring that at least one rotation axis is defined by the pelvic markers. The shank marker represented an initial but arbitrary guess for the plane, which was then subsequently rotated using a controller that was created and parented underneath the pelvis locator, allowing only rotation around the axis between the two markers (green axis) (Supplementary Figure S1B). Identical to the six marker IK setup, an IK handle was created between the hip and ankle joint, with a pole-vector constraint, guided by the shank markers. The controller was then used to ‘rotoscope’ the pelvis into position and match it to the X-ray shadows by rotating it around the axis defined by the two remaining pelvis markers, thus ensuring that the hip joint was placed correctly within the 3D space and the hip and knee joint rotations were calculated through the IK solver.

**Four marker IK rig setup (Evaluation Rig 2)**

- *Maya joint marionette creation*: It followed the same procedure as DDNC04 and DDNC10 hindlimb setup.
- *IK rig setup:* Due to the two missing markers, an additional set of controls had to be introduced. Firstly, the hip controller which determined the position of the pelvis around the axis of the two remaining pelvis markers (green axis) and secondly, the shank controller which determined the position of the ankle and knee joints around the axis of the two tibia markers (purple axis) (Supplementary Figure S1C). The hip controller was identically setup as in the five marker IK rig setup and the shank controller in similar fashion. An additional locator was created and point-constrained to both tibia markers with a weighting factor of 0.5 each. It was subsequently aim-constrained at one of the tibia markers with one of the pelvis markers as *World Up Object*, to represent an initial guess for the rotation plane around axis through both tibia markers. The controller was parented underneath this shank locator and only allowed to rotate around the axis through the tibia markers (purple axis). Instead of parenting the IK handle and the Knee_AIM_LOC directly underneath the locator, as in the six marker IK rig setup, they were parented underneath the controller, thus constraining their position to a circle around the axis between the markers (purple axis) controlled through rotation of the shank control. Both the hip controller and shank controller where then manually animated to rotoscope the pelvis and shank and to match the bones to their X-ray shadows.

**Three marker IK rig setup (Evaluation Rig 3 and all forelimbs):**

- *Maya joint marionette creation:* We followed the same procedure as the DDNC04 and DDNC10 hindlimb setup. The setup for the forelimb was identical to the hindlimb (which is discussed below), however the forelimb’s hierarchical order was instead as follows: Pectoral Girdle > Shoulder > Elbow > Wrist > (Third) Metacarpophalangeal Joint > (Third) Finger_Tip.
- *IK rig set up:* Due to the lack of an appropriate number of markers to automatically position and orient the skeletal elements into place, a number of additional controls had to be created. While the long-axis of the forearm was defined by the axis through the proximal and distal tibia markers, the position of the knee joint could only be narrowed down to a circle around the axis through the tibia markers. Additionally, the position of the hip joint was even less constrained, potentially being positioned anywhere on a sphere around the pelvis marker. Therefore two IK joint marionettes, controlled by three controllers, were created to constrain and guide the position of the knee, hip joint and pelvic girdle based on the tracked marker positions.
  - Firstly, the shank controller defined the knee position by rotating the shank around the tibia marker axis (purple axis).
  - Secondly, the hip joint controller controlled the hip position by rotating it around the axis from the knee joint to the pelvis marker (salmon coloured axis). This allowed calculating the hip and knee rotations, as the knee was restricted to only allow movement around the Z-axis.
  - Thirdly, the pelvis orientation was controlled by a third controller, rotating it around the axis from the hip jpint to the pelvis marker (green axis).
- The axis upon which the hip controller acted was established by creating a locator (Knee_POS_LOC) at the position of the Knee joint and parenting it underneath the shank controller (Shank_Rot_CTRL). A second locator (Hip_CTRL_POS_LOC) was then created and point constrained to the pelvis marker and the Knee_POS_LOC with a weighting of 0.5 each (Supplementary Figure S2). Thus ensuring that it was always positioned between the two reference points. It was subsequently aim-constrained at the pelvis marker using one of the tibia markers as *World Up Object*. The hip controller (Hip_Rot_CTRL) was parented underneath the Hip_CTRL_POS_LOC and only allowed rotation around the axis between the two reference points. A third locator was then created (Hip_AIM_LOC) and placed at the hip joint position in the reference pose and parented underneath the hip controller (Supplementary Figure S2). Thus, rotation of the hip controller resulted in rotation of the Hip_AIM_LOC around the axis between the knee joint and the pelvis marker (salmon coloured axis; Supplementary Figure S1D).
- Following the creation of the hip controller an additional three-joint IK rig was created (IK_CTRL_RIG; i.e., see salmon coloured joint marionette in Supplementary Figure S1D) to determine the hip joint position. In the reference pose the first joint was positioned and point constrained to the pelvis marker, the second joint was positioned onto the hip joint and the third and final joint was placed onto the knee joint. The first and last joints were connected with an IK handle with a rotate plane solver, which was then parented underneath the hip controller and pole vector constrained to the Hip_AIM_LOC (Supplementary Figure S2), thus ensuring that when the hip controller was rotated the hip joint followed.
- After establishing the ankle, knee and hip joints, the pelvic girdle remained last to be determined, so an additional controller was created for this purpose. To determine the position of the controller, a locator (Pelvis_CTRL_POS_LOC) was created and point-constrained to the pelvis marker and the IK_CTRL_RIG hip joint with a weighting of 0.5 each and was subsequently aim-constrained at the pelvis marker with the Hip_CTRL_POS_LOC as *World Up Object* (Supplementary Figure S2). The pelvis controller (Pelvis_Rot_CTRL) was parented underneath the Pelvis_CTRL_POS_LOC and the rotation was restricted to around the axis between hip joint and the pelvis marker (green axis; Supplementary Figure S1).
- To align the pelvis joint of the IK rig and the IK_CTRL_RIG, an additional helper was created and placed at the pelvis joint position in the reference pose and hierarchically parented underneath the pelvis controller (Supplementary Figure S2; in Supplementary Figure S2 the helper is a *Joint* object, however, a *Locator* could be used instead). The pelvis joint of the initial IK rig (i.e., see purple joint marionette in Supplementary Figure S1D) was then parent-constrained to this helper, thus ensuring that the hip joint aligned with the IK_CTRL_RIG hip joint and the other downstream joints aligned and were correctly calculated through the IK solver. The setup for the forelimb is neither figured nor described directly herein, however, it was identical to the described process for the hindlimb.


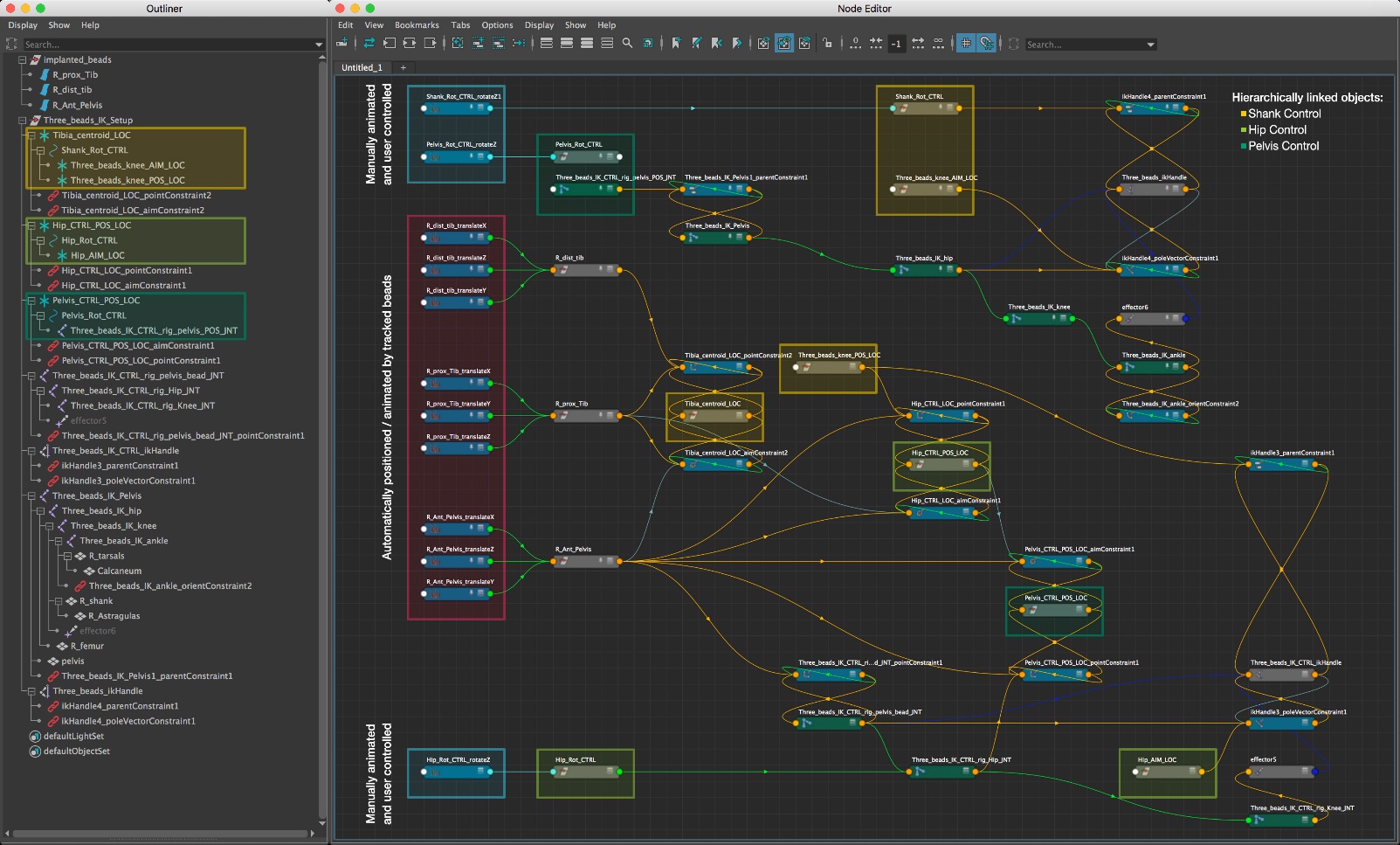


**Supplementary Figure S2.** Annotated *Outliner* and *Node Editor* highlighting the relationships, constraints and implementation of the three-marker IK setup in Maya. While the components of the controller setup are not connected directly in the *Node Editor* (right), they are nested hierarchically in the *Outliner* (left), therefore, transformations of the parent are automatically passed on to their respective children. Note how the positions of the *Locators* under which the control curves are nested are always constrained by a *point constraint* (to ensure their correct position) and an *aim constraint* (to ensure their consistent orientation). Outlined boxes: red, automatically keyed translation animation nodes for the tracked bead position (positions imported from a CSV file through the XROMM tool shelf); blue, manually keyed rotations animation nodes; yellow, helper objects connected to the shank control; light green, helper objects connected to the hip control; dark green, helper objects connected to the pelvis control. Note the animation nodes are automatically generated when an attribute is keyed.

The result of all rigs (hindlimbs and forelimbs) was a set of XROMM-informed bony motions from which the rotations of each joint and the translations and rotations of the pelvic/pectoral girdles were exported. These translations and rotations can be used to animate musculoskeletal models or conduct simulations from which we can extract biomechanical information (e.g., Wiseman et al. 2021). Because additional rotoscoping was required to move the manus/pes into position (DDNC04 and DDNC09) and to place the pectoral girdle in position (all forelimbs), we acknowledge that this process is a combination of scientific rotoscoping and IK solver-methodology and, therefore, we call this process *IK marker-guided rotoscoping*.


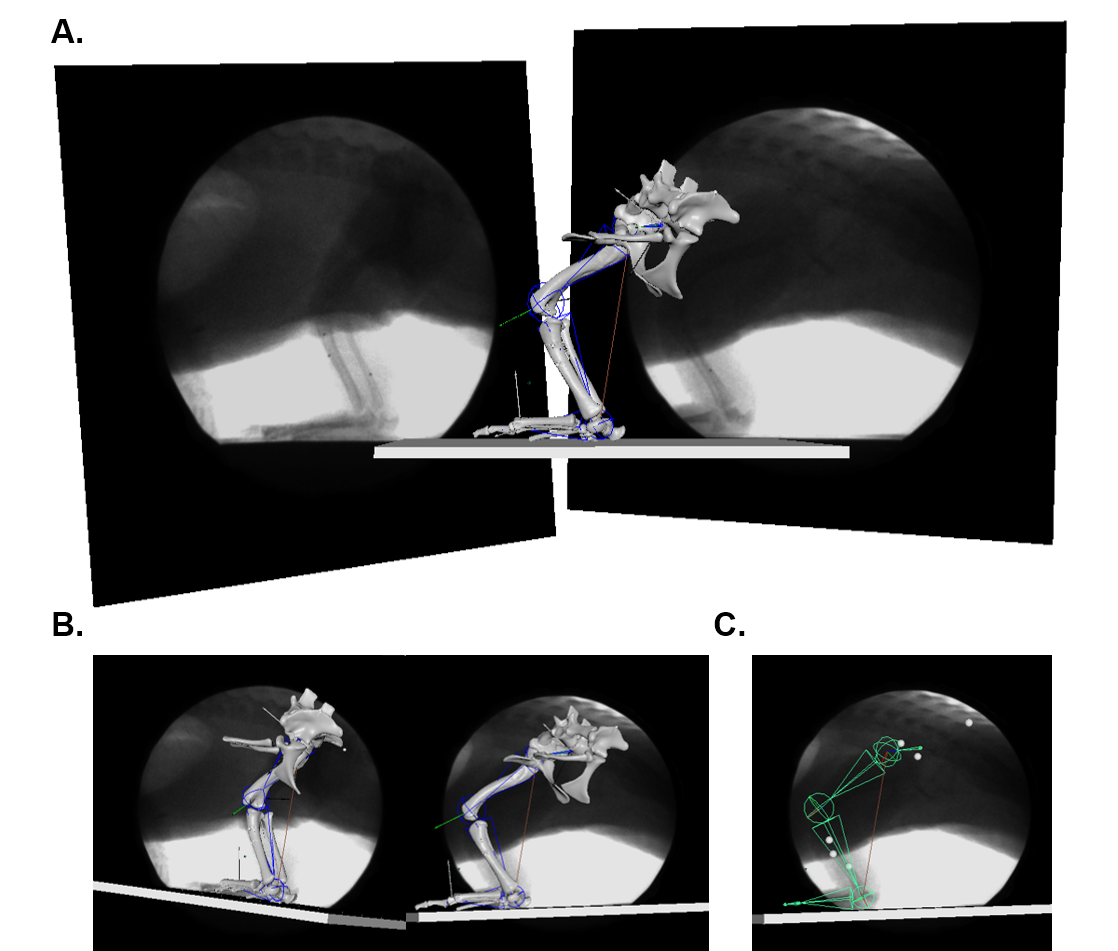


**Supplementary Figure S3.** XROMM was used to capture crocodile movement across a flat surface, with six markers placed in the hindlimb (DDNC04). An IK rig was used to track the movement, shown in blue in panels A and B. The floor was created as a 3D object to assist with aligning the pes and digit segments, which did not have any markers implanted and were rotoscoped into position via rotation around the *z-*axis only. This floor is shown in white towards the bottom of the view in each panel. (A) 3D view of the crocodile walking. (B) Each camera’s view of the movement. (C) One camera’s view of the movement showing just the rig highlighted in green, with each marker emphasised in white.
